# A Preliminary Study on the Concentration of Oxytetracycline and 4-Epi-Oxytetracycline in Sow Milk

**DOI:** 10.3390/molecules27103258

**Published:** 2022-05-19

**Authors:** Piotr Cybulski, Anna Gajda, Małgorzata Gbylik-Sikorska, Artur Jabłoński

**Affiliations:** 1 Goodvalley Agro S.A., Dworcowa 25, 77-320 Przechlewo, Poland; piotr.cybulski@goodvalley.com; 2 Department of Pharmacology and Toxicology, National Veterinary Research Institute, Partyzantow 57, 24-100 Pulawy, Poland; malgorzata.gbylik@piwet.pulawy.pl; 3 Center of Translational Medicine, Faculty of Veterinary Medicine, Warsaw University of Life Sciences, Nowoursynowska 100, 02-787 Warsaw, Poland

**Keywords:** antibiotics, oxytetracycline, 4-epi-oxytetracycline, milk, sows, UHPLC–MS/MS

## Abstract

Even though modern analytical chemistry has developed a methodology enabling evaluation of the presence of OTC in milk, data regarding its concentration in the material collected from lactating sows are missing. Therefore, this paper was intended to provide new data on the transmission of OTC and its epimer, 4-epi-oxytetracycline (4-epi-OTC), in the milk of lactating sows after a singular intramuscular administration of a long-acting form of the antibiotic. The determination of OTC and 4-epi-OTC was carried out using ultrahigh-performance liquid chromatography with mass spectrometry (UHPLC–MS/MS). The highest average concentration of antibiotic (1132.2 µgL^−1^) was observed in samples collected 1 day after the administration of the drug. The average OTC level at day 3 was 358 µgL^−1^. The average concentration of the antibiotic found on the 21st day was 12.3 µgL^−1^. The highest average concentration of 4-epi-OTC—i.e., 54 µgL^−1^—was noted 1 day after the administration. Amongst samples collected at day 3, the average level of the substance in question was 26.4 µgL^−1^. The average value observed at day 21 was 1.5 µgL^−1^. Our results indicated considerable OTC and 4-epi-OTC transmission into the milk of lactating sows.

## 1. Introduction

The group of tetracycline antibiotics is widely applied in modern veterinary medicine [1,2,3]. One of these antibiotics is oxytetracycline (OTC)—a product of the metabolism of *Streptomyces rimosus.* Essential characteristics of OTC, including its mode of action (i.e., inhibition of protein synthesis through reversible binding to the 30S subunit of the ribosome), bacteriostatic activity against a wide spectrum of pathogenic organisms, and its easy availability in both conventional and long-acting injectable formulations, collectively make this antibiotic particularly useful for the treatment of a great number of diseases in animals, including food-producing species [4]. Therefore, OTC is routinely used in veterinary practice in ruminants [5,6,7], swine [8,9], poultry [10,11,12], and aquaculture [13,14]. Additionally, this antibiotic is used for therapy in less obvious food-producing animal species, such as the honey bee [15,16] or horses [17,18].

Since animal byproducts are an important source of essential nutrients for humans, numerous studies have presented harmful effects of dietary exposure to residues of antibiotics, including OTC, on the final consumer [19,20]. In general, total world production of the four most consumed types of animal meat—i.e., chicken, pork, beef, and mutton—is estimated at 339 Mt per annum, whereas global cow’s milk production is approximately 874 Mt. Moreover, the production of milk is anticipated to grow steadily at 1.7% per annum over the next decade [21]. As a far-reaching consequence of the aforementioned share of the main agricultural commodities, the vast majority of the complete pharmacokinetic–pharmacodynamic studies of OTC have been performed using cattle as a model [22,23,24,25]. In fact, studies developing methods of qualitative and quantitative screening of milk for the presence of antibiotics are driven by frequently reported milk contamination—mostly in developing countries [26,27,28]. Thus, this problem is much less explored in other species. Even though modern analytical chemistry has a proper, validated methodology [29,30], peer-reviewed publications describing concentrations of OTC in monogastric animals’ milk—including sows—have not been published to date.

Today, modern and systematic approaches to swine health management require intimate knowledge about the long-term effects of antibiotic usage in swine herds. Such an understanding should allow researchers to recognise and systematically analyse all possible on-farm antimicrobial resistance patterns. Data dealing with the concentrations of some antibiotics in sow milk, and the potential influence of prolonged exposure to them at subtherapeutic doses on suckling piglets, seem to be a missing link in modern knowledge. Therefore, the aim of our research was to determine the concentrations of OTC and its epimer, 4-epi-oxytetracycline (4-epi-OTC), in milk samples collected from highly prolific sows treated at the early stage of lactation with a single intramuscular injection of long-acting form of the antibiotic.

## 2. Result

### 2.1. Optimisation and Validation of Analytical Methods

**Figure 1:**
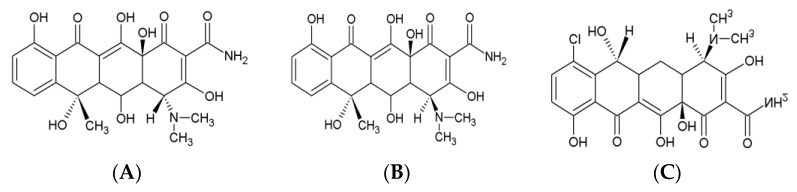
Chemical structures of (**A**) OTC, (**B**) 4-epi-OTC, and (**C**) DMC.

In the optimisation of extraction step, comparative studies of different extraction solutions were conducted. To isolate OTC and 4-epi-OTC from milk, the oxalic acid buffer, 5% trichloroacetic acid, and acetonitrile were tested. OTC and 4-epi-OTC were isolated with low recovery when acetonitrile was used. With oxalic acid buffer, good results were obtained, but a further clean-up step with SPE columns was needed. After treatment of milk samples with a 5% solution of TCA, satisfactory recoveries were obtained, with PVDF filters for the clean-up. The simple and fast extraction procedure with the usage of 5% trichloroacetic acid was found to be an accurate, labour-efficient method allowing for the analysis of many milk samples in a very short time. During the optimisation of OTC and 4-epi-OTC separation and LC–MS/MS analysis, different compositions of the mobile phase were studied. To minimise peak tailing, acidic mobile phases are generally used in the analysis of tetracyclines. In this study, oxalic acid in a mobile phase was used, as it shows a significant efficiency in softening the effect of residual silanols on the stationary phase, and good OTC and 4-epi-OTC separation, without tailing. However, it should be taken into account that mobile phases containing non-volatile compounds used in LC–MS/MS may cause clogging at the interface and a build-up of deposits at the ion source. In the next step, various percentages of ACN with formic acid were tested, but the separation was not satisfactory. Finally, the mobile phase consisting of 0.1% formic acid in acetonitrile and 0.1% formic acid in water used in this method allowed us to obtain sharp and symmetrical peaks of OTC and 4-epi-OTC without any clogging problems, with an analyte peak asymmetry factor close to 1.0. TCs give (M+H)^+^, (M+H−NH_3_)^+^, and (M+H−NH_3_−H_2_O)^+^ ions in the ESI mass spectra, and these ions are very useful for the confirmation of these compounds. The fragmentation pathways of TCs were also described by Kamel et al. [31], and were mainly explained by the loss of H_2_O, NH_3_, CO, and NH(CH_3_)_2_. Thus, the most abundant product ions were chosen as the quantification ions. For the detection of OTC and 4-epi-OTC in milk samples, the first transition 461 → 426 was used, while for confirmation the second transition 461 → 443 was applied. The chemical structures of OTC, 4-epi-OTC, and demeclocycline as an internal standard are presented in Figure 1.

**Figure 2:**
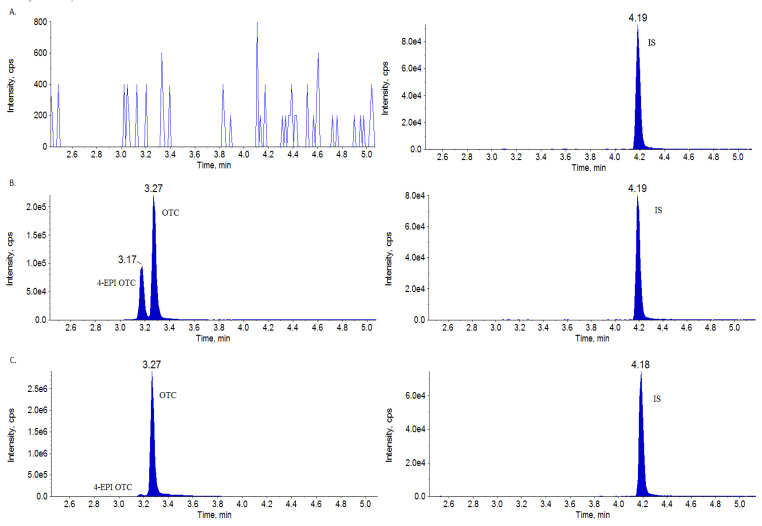
Ion chromatograms (transition for OTC and 4-epi-OTC: 461 → 426 and IS: 465 → 448) of (**A**) blank milk sample; (**B**) OTC and 4-epi-OTC milk sample fortified at 100 µgL^−1^; (**C**) milk sample obtained 1 day after the intramuscular injection of veterinary medicinal product with OTC at a concentration of 1120 µgL^−1^.

The development and validation of an analytical method for the determination of OTC and 4-epi-OTC in sow milk were successfully accomplished, performed with high accuracy and precision. During the validation process, all matrix-matched calibration curves showed good linearity (r^2^ > 0.999). No matrix interferences were observed in the retention time of the target analyte in milk samples. The chromatographic analysis time was short, with OTC and 4-epi-OTC as sharp and symmetrical peaks with no interference. Figure 2 presents the chromatograms of a blank milk sample, a milk sample fortified with OTC and 4-epi-OTC at the level of 100 µgL^−1^, and a sample obtained on the 1st day following the intramuscular injection of the antibiotic. The validation results obtained for the presented method were repeatable (RSDs lower than 10%) and reproducible (RSDs lower than 15%), indicating good precision of the method, with repeatability relative standard deviations (RSDs) in the range of 5.0–6.8% for OTC and 3.2–5.3% for 4-epi-OTC, at all fortification levels. The intra-laboratory reproducibility RSDs for OTC were calculated in the range of 6.4–9.5% for OTC and 6.2–8.3% for 4-epi-OTC. The recovery was calculated in the range of 95.7–103.1% for OTC and 97–103.3% for 4-epi-OTC, depending on the fortification level. The validation results are presented in Table 1. Satisfactory sensitivity was achieved, with LOD = 2 µgL^−1^ and LOQ = 5 µgL^−1^ for both compounds. In the stability testing, OTC and 4-epi-OTC were stable for 1 month, with the ratio M_−19 °C_/M_fresh_ of 1 after 1, 2, 3, and 4 weeks.

### 2.2. Detection and Quantification of OTC

The concentration of OTC in all 30 milk samples, collected 6 times from 5 sows between day 1 and day 21 after the intramuscular injection of the antibiotic, was above the LOQ, with the highest average concentration of antibiotic being 1132.2 µgL^−1^ 1 day after drug administration, and the variation in individual animals ranging from 581 to 1380 µgL^−1^ (Table 2). The average OTC level at day 3 was 358 (189–803) µgL^−1^. The average concentration of the antibiotic found on the following days, i.e., 5, 7, 14, and 21, was 173.8 µgL^−1^, 178.6 µgL^−1^, 50 µgL^−1^, and 12.3 µgL^−1^, respectively.

### 2.3. Detection and Quantification of 4-Epi-OTC

The concentration of 4-epi-OTC in all of the analysed milk samples was above the LOQ (Table 3). Similarly to OTC, the highest average concentration of 4-epi-OTC—i.e., 54 µgL^−1^, with deviation between individuals ranging from 36.8 µgL^−1^ to 62.6 µgL^−1^—was observed 1 day after the treatment. Amongst samples collected on day 3, the average level of the substance in question was 26.4 (13.3–60.9) µgL^−1^. The average values observed on days 5, 7, 14, and 21 were 14.9 µgL^−1^, 15.7 µgL^−1^, 5 µgL^−1^, and 1.5 µgL^−1^, respectively.

## 3. Discussion

OTC is a lipophilic and relatively small molecule that can easily pass through cell membranes; thus, as seen from our investigation, a single intramuscular administration of a long-acting form of the antibiotic in lactating sows results in considerable transmission of OTC and 4-epi-OTC into their milk. The highest average concentration of OTC (i.e., 1132.2 µgL^−1^, with variation in individual animals from 581 µgL^−1^ to 1380 µgL^−1^) was observed in milk samples collected 1 day after drug administration. The value regarding the concentration of its epimer, 4-epi-OTC, noted at the same time point was 54 (36.8–62.6) µgL^−1^.

The available publications referring to the pharmacokinetics of OTC in swine thoroughly describe its activity after intravenous [32], intramuscular [33], and oral administration [33,34]; however, the aforementioned studies were focused on blood, tissue, or oral fluid concentrations of the antibiotic [9,35]. The penetration of OTC into the milk of lactating sows has not yet been explored. One possible explanation of such a phenomenon is a complete lack of—or rather marginal in some cases—a role of monogastric animals’ milk in human consumption [36].

Pigs are often used as the primary model for biomedical sciences; thus, extrapolation of food safety norms for human consumption allows us to draw preliminary conclusions regarding possible impact of contaminated milk on suckling piglets’ health. According to the Commission Regulation (EU) No 37/2010 of 22 December 2009 on pharmacologically active substances and their classification regarding maximum residue limits in foodstuffs of animal origin, as well as the Codex Alimentarius [37], the maximum residue limit (MRL) of OTC and 4-epi-OTC combined in bovine milk is 100 µgL^−1^, with the acceptable daily intake (ADI) estimated at 0–30 µg/kg of body weight. Therefore, assuming that the body weight of a typical adult human is 70 kg, theoretical consumption of bovine milk (containing the highest legal OTC concentration) that still does not lead to appreciable health risk is up to 21 litres per day over an entire lifetime. Results estimated using the aforementioned ADI reference value and the concentrations of OTC in the samples of sow milk obtained in our study indicate that a typical 2-day-old suckling piglet may be exposed to an OTC intake over 10-fold greater than the limits set by the official standards presented by the authorities (Table 4). 

Generally, tetracyclines are known for their low degree of toxicity, resulting from the poor absorption from the gastrointestinal tract; nevertheless, taking into account relatively long-term exposure during an early period of bacterial colonisation, such a phenomenon may irreversibly alter the local microbiota of suckling piglets, and lead to potential health issues in the following weeks of life. The possible negative influence on piglets consuming milk contaminated with OTC or other antimicrobials has not yet been investigated, and merits further clinical investigation.

## 4. Materials and Methods

### 4.1. Animal Experiment and Sample Collection

This study was performed in July 2021 in a high-performing sow farm (8000 DanBred sows) located in northern Poland. The animals were housed in a weekly farrowing system on a slatted floor, and received wheat- and barley-based lactation pelleted feed, fed from the 2nd week before the date of expected farrow. The levels of protein, fibre, and fat were 16.1%, 4.8%, and 5.5%, respectively. All of the animals were reared under conditions meeting the requirements of Council Directive 2008/120/EC of 18 December 2008, laying down the minimum standards for the protection of the pigs.

The subjects of the investigation were 5 multiparous sows treated with long-acting injectable OTC 1 day after farrow because of injuries caused by labour dystocia. All of the pigs were injected into the neck intramuscularly by a veterinarian using Tetradur LA-300 (Merial S.A.S., Lyon, France) at a single dose of 30 mg per kg of body weight. Prior to the drug’s administration, all of the animals were tested for the presence of OTC in the milk, and achieved negative results.

In order to assess the OTC concentrations in milk, the samples were collected manually by the same veterinarian in the same hour at 6 specified time points, i.e., 1, 3, 5, 7, 14, and 21 days post-injection. Each sample, containing 25 mL of milk, was collected using a sterile plastic screw-cap specimen jar, cooled, and then stored at −19 °C until the laboratory analysis. UHPLC–MS/MS analysis was performed 2 days after each milk collection.

### 4.2. Quantitative Analysis by UHPLC–MS/MS

#### 4.2.1. Reagents and Chemicals

All reagents used were of an analytical grade. Reference standards of OTC, 4-epi-OTC, and demeclocycline (DMC) as an internal standard (IS) were obtained from LGC Standards (Teddington, Middlesex, UK). Trichloroacetic acid (TCA) was purchased from Sigma-Aldrich (St. Louis, MO, USA). Acetonitrile and methanol were from J.T. Baker (Deventer, the Netherlands). Formic acid was from Fluka (St. Louis, MO, USA). Syringe 0.22 μm hydrophilic polyvinylidene fluoride (PVDF) membrane filters were provided by Restek (College, PA, USA).

Individual stock standard solutions (1000 µg/mL) for OTC, 4-epi-OTC, and DMC were prepared in methanol and stored in polypropylene vessels. Individual stock standard solutions were stable for at least 6 months when retained in a dark place at 18 °C. The standard working solutions were obtained by diluting the standard stock solutions using ultrapure water, and stored in a refrigerator at 4–8 °C for 1 month.

#### 4.2.2. UHPLC–MS/MS Analysis

The determination of OTC and 4-epi-OTC in porcine milk was carried out via ultrahigh-performance liquid chromatography with mass spectrometry (UHPLC–MS/MS) on a Shimadzu Nexera X2 UHPLC system (Shimadzu, Kyouto, Japan) connected to a SCIEX 4500 triple-quadrupole mass spectrometer (Sciex, Framingham, MA, USA). Analyst 1.6.2 software (SCIEX, Framingham, MA, USA) was used to process the data and control the LC–MS/MS system. The mass spectrometry detection was operated in the positive ESI mode, with the MS data acquisition in the multiple reaction monitoring (MRM) mode. The following precursor → product ion pairs were monitored: OTC: 461 → 426/443 and DMC 465 → 448. Nitrogen was used as a collision gas, curtain gas, and nebuliser gas.

The operating parameters were set as follows: curtain gas (N_2_): 20; nebuliser gas (N_2_): 50; collision gas (N_2_): medium; auxiliary gas: 60; ion spray voltage: 5500 V, temperature: 400 °C. The MS/MS parameters for OTC were established as follows: declustering potential (DP): 50 V, cell exit potential (CXP): 13 V, entrance potential (EP): 10 V, collision energy (CE) for ion 1 and ion 2: 27 and 17 V, respectively. For DMC as an IS, the following values were set: DP = 100 V, CE = 15 V, CXP = 15 V and EP = 10 V. The LC separation was achieved using a Luna C18 column, 50 mm × 2.0 mm × 3.0 μm (Phenomenex, Torrance, CA, USA), with a 2 × 4 mm guard column of the same material (Phenomenex, Torrance, CA, USA). The oven temperature was set to 35 °C. The mobile phase composition was as follows: solvent A—0.1% formic acid in water, and solvent B—0.1% formic acid in acetonitrile. The applied gradient was 0–5.0 min 5% solvent B; 5.01–6.34 min increased to 80% solvent B, and then 6.35–8.00 min decreased to 5% solvent B, operating at a flow rate of 0.45 mL/min. The injection volume was 10 μL.

#### 4.2.3. Sample Preparation 

For each of the milk samples to be analysed, an aliquot of 2 mL was placed into a polypropylene centrifuge tube, and the internal standard was added at 2 µg/mL. Next, 6 mL of 5% TCA was added, stirred for 10 min, and centrifuged for 10 min at 3396 × rcf. Then, about 1 mL of supernatant was filtered through a 0.22 mm PVDF filter into a vial and analysed using the LC–MS/MS instrument.

#### 4.2.4. Analytical Method Validation 

The developed method was validated according to the recommendations of the Commission Regulation (EU) 2021/808 of 22 March 2021 on the performance of analytical methods for residues of pharmacologically active substances used in food-producing animals, and on the interpretation of results as well as on the methods to be used for sampling and repealing Decisions 2002/657/EC and 98/179/EC. The validation pack included the following parameters: linearity, selectivity/specificity, precision (expressed as repeatability and intra-laboratory reproducibility), and recovery. Additionally, the limit of quantification (LOQ) was evaluated as the lowest level of the matrix-matched calibration curve. The limit of detection (LOD) was calculated in relation to S/N = 3 on the chromatograms of blank milk samples. The linearity was evaluated via two matrix-matched calibration curves prepared in the concentration ranges of 5–300 µgL^−1^ and 500–1500 µgL^−1^. Precision was established at each fortification level by evaluating relative standard deviation (RSD, %). Repeatability was calculated after analysis of 6 milk samples spiked with OTC and 4-epi-OTC at 4 concentrations—5, 50, 100, and 150 µgL^−1^—by the same operator, on the same day, with the same instrument. The intra-laboratory reproducibility was determined in the same way as the repeatability, by different operators analysing another 2 sets of 6 spiked samples on 2 different days. Based on these spiked samples’ replicates, as with the precision, the average recovery was studied. The mean concentrations of the analytes in the fortified samples in relation to the matrix-matched calibration curves were compared. The specificity of the method was determined via the repeated injection of 10 milk samples. In the stability testing, for the purposes of the conducted experiment, blank milk was divided into five aliquots, and each aliquot was fortified with OTC and 4-epi-OTC at the level of 100 µg/kg. Right after the preparation of the samples, 1 aliquot was analysed in 10 replicates. The remaining aliquots were stored at −19 °C and analysed after 1, 2, 3, and 4 weeks (*n* = 10). The mean values of the freshly prepared samples (M_fresh_) were compared with the mean values of samples stored at −19 °C (M_−19 °C_) for a specific period of time. Fortified samples were considered sufficiently stable when the ratio M_−19 °C)_/M_fresh_ was between 0.80 and 1.20.

## 5. Conclusions

To the best of our knowledge, the results obtained in this study are the very first describing the concentration of OTC in milk samples collected from lactating sows treated with a single intramuscular injection of a long-acting form of the antibiotic. Moreover, our results demonstrate the high utility of porcine milk as a medium for the detection of OTC; its application to pharmacokinetic studies in pigs and possible influence on future research aimed at increasing the microbiological safety of food-producing animals are exceptionally valuable. Additionally, crucial factors responsible for the deviation observed between the individuals and its impact on suckling piglets’ health are not yet understood, and merit further clinical research.

## Figures and Tables

**Table 1:** Validation parameters (precision and recovery) of the method for the determination of OTC and 4-epi-OTC in sow milk.

Analyte	Fortification Level (µgL^−1^)	Repeatability (%)	Reproducibility (%)	Recovery (%)
OTC	5.0	5.0	6.4	101.8
	50.0	7.4	9.5	99.0
	100	6.8	8.5	95.7
	150	5.8	7.9	103.1
4-epi-OTC	5.0	5.0	8.3	103.3
	50.0	5.3	7.2	98.0
	100	3.2	8.1	87.8
	150	4.8	6.2	97.1

**Table 2:** Concentrations of oxytetracycline (OTC) in sow milk after a single intramuscular administration of a long-acting form of the drug at a single dose of 30 mg per kg of body weight.

Time (days)	Sow 1	Sow 2	Sow 3	Sow 4	Sow 5	Min.	Max.	Av.	SD
OTC concentration in milk (µgL^−1^)									
1	1120	1380	581	1220	1360	581	1380	1132	326
3	354	803	252	192	189	189	803	358	257
5	188	132	168	150	231	132	231	173	38.1
7	196	75.8	116	81.4	424	76.0	724	178	145
14	35.6	37.2	72.2	44.4	60.5	36.0	61.0	50.0	15.9
21	9.3	4.4	22.3	15.7	9.8	4.0	22.0	12.3	6.9

**Table 3:** Concentrations of 4-epi-oxytetracycline (4-epi-OTC) in sow milk after a single intramuscular administration of long-acting oxytetracycline (OTC) at a single dose of 30 mg per kg of body weight.

Time (days)	Sow 1	Sow 2	Sow 3	Sow 4	Sow 5	Min.	Max.	Av.	SD
4-epi-OTC concentration in milk (µgL^−1^)									
1	62.6	61.0	36.8	58.6	51.1	36.8	62.6	54.0	10.6
3	28.7	60.9	15.3	14.0	13.3	13.3	60.9	26.4	20.3
5	18.6	10.8	18.7	12.4	13.8	10.8	18.7	14.9	3.6
7	20.6	7.2	7.9	7.7	35.2	7.2	35.2	15.7	12.3
14	4.4	4.4	4.8	5.0	6.3	4.4	6.3	5.0	0.8
21	1.3	0.7	2.4	2.2	1.1	0.7	2.4	1.5	0.7

**Table 4:** Estimated exposure of suckling piglets to oxytetracycline (OTC) ingested with contaminated sow milk after a single intramuscular administration of a long-acting form of the drug given to sows at a single dose of 30 mg per kg of body weight.

Time Post-Administration	Age of Piglets	Average OTC Concentration in Sow Milk [This Study]	Estimated Weight of an Average Piglet [38]	Acceptable Daily OTC Intake Per Piglet ^1^	Estimated Daily Milk Intake [39]	Acceptable Intake of OTC Contaminated Milk	Estimated Intake of Contaminated Milk to Acceptable Intake of Contaminated Milk
days	days	µgL^−1^	kg/piglet	µg/Kgbw^−1^	L/piglet	L/piglet	ratio
*a*	*b* = (*a* + 1)	*c*	*d*	*e* = (*D* × 30)	*f*	*g* = (*e*:*c*)	*i* = (*f*:*g*)
1	2	1132.2	1.28	38	0.35	0.03	10.32
3	4	358.0	1.82	55	0.64	0.15	4.20
5	5	173.8	2.36	71	0.64	0.41	1.57
7	8	178.6	3.16	95	0.90	0.53	1.70
14	15	50.0	5.05	152	0.99	3.03	0.33
21	22	12.3	6.93	208	1.05	16.94	0.06

^1^ Extrapolation of food safety norms for human consumption.

## Data Availability

Data are contained within the article.

## References

[1] Mitema E.S., Kikuvi G.M., Wegener H.C., Stohr K. (2001). An assessment of antimicrobial consumption in food producing animals in Kenya. J. Vet. Pharmacol. Therapeutics.

[2] Kariuki S., Onsare R., Mwituria J., Ng’etich R., Nafula C., Karimi K., Karimi P., Njeruh F., Irungu P., Mitema E. (2013). FAO/WHO Project Report: Improving Food Safety in Meat Value Chains in Kenya. Food Protection Trends. https://www.fao.org/documents/card/fr/c/8b811eb5-f02c-448e-b288-4b5a6d2cb040/.

[3] Attuabi M., Borck Høg B., Müller-Pebody B. (2020). DANMAP 2020 Use of Antimicrobial Agents and Occurrence of Antimicrobial Resistance in Bacteria from Food Animals, Food and Humans in Denmark. https://www.danmap.org/reports/2020.

[4] Riviere J.E., Papich M.G. (2018). Tetracycline Antibiotics. Veterinary Pharmacology and Therapeutics.

[5] Furusawa F. (1999). Rapid liquid chromatographic determination of oxytetracycline in milk. J. Chromatogr. A.

[6] Payne M.A., Babish J.G., Bulgin M., Lane M., Wetzlich S., Craigmill A.L. (2002). Serum pharmacokinetics and tissue and milk residues of oxytetracycline in goats following a single intramuscular injection of a long-acting preparation and milk residues following a single subcutaneous injection. J. Vet. Pharmacol. Ther..

[7] Cinquina A.L., Longo F., Anastasi G., Giannetti L., Cozzani R. (2003). Validation of a high-performance liquid chromatography method for the determination of oxytetracycline, tetracycline, chlortetracycline and doxycycline in bovine milk and muscle. J. Chromatogr. A.

[8] Cherlet M., Schelkens M., Croubels S., Backer P.D. (2003). Quantitative multi-residue analysis of tetracyclines and their 4-epimers in pig tissues by high-performance liquid chromatography combined with positive-ion electrospray ionization mass spectrometry. Anal. Chim. Acta.

[9] Gajda A., Jablonski A., Sikorska M., Posyniak A. (2017). Correlation between oral fluid and plasma oxytetracycline concentrations after intramuscular administration in pigs. J. Vet. Pharmacol. Ther..

[10] Gajda A., Nowacka-Kozak E., Gbylik-Sikorska M., Posyniak A. (2019). Multi-residues UHPLC–MS/MS analysis of 53 antibacterial compounds in poultry feathers as an analytical tool in food safety assurance. J. Chromatogr. B.

[11] Manu G.C., Chinnu M.V., Ramnath V., Rajan S. (2019). Oxytetracycline Residue Analysis in Poultry Meat by Ultra High Performance Liquid Chromatography. Vet. Res. Int..

[12] Puvača N., Lika E., Tufarelli V., Bursić V., Pelić D.L., Nikolova N., Petrović A., Prodanović R., Vuković G., Lević J. (2020). Influence of Different Tetracycline Antimicrobial Therapy of Mycoplasma (Mycoplasma synoviae) in Laying Hens Compared to Tea Tree Essential Oil on Table Egg Quality and Antibiotic Residues. Foods.

[13] Coyne R., Bergh Q., Samuelsen O.B. (2004). One-step liquid chromatographic method for the determination of oxytetracycline in fish muscle. J. Chromatogr. B.

[14] Wongtangprasert T., Natakuathung W., Pimpitak U., Buakeaw A., Palaga T., Komolpis K., Khongchareonporn N. (2014). Production of a monoclonal antibody against oxytetracycline and its application for oxytetracycline residue detection in shrimp. J. Zhejiang Univ. Sci. B.

[15] Gajda A., Posyniak A., Bober A., Bladek T., Zmudzki J. (2013). Oxytetracycline residues in honey analyzed by liquid chromatography with UV detection. J. Api. Sci..

[16] Ortiz-Alvarado Y., Clark D.R., Vega-Melendez C.J., Flores-Cruz Z., Domingez-Bello M.G., Giray T. (2020). Antibiotics in hives and their effects on honey bee physiology and behavioral development. Biol. Open.

[17] Pilloud M. (1973). Pharmacokinetics, plasma protein binding and dosage of oxytetracycline in cattle and horses. Res. Vet. Sci..

[18] Jansen M.L. (1988). Oxytetracycline by injection for horses. N. Z. Vet. J..

[19] Nisha A.R. (2009). Antibiotic residues—A global health hazard. Vet. World.

[20] Oka H., Ito Y., Matsumoto H. (2000). Chromatographic analysis of tetracycline antibiotics in foods. J. Chromatogr. A.

[21] (2019). OECD-FAO Agricultural Outlook 2019−2028. https://www.oecd.org/agriculture/oecd-fao-agricultural-outlook-2019/.

[22] Landoni M.F., Errecalde J.O. (1992). Tissue concentrations of a long-acting oxytetracycline formulation after intramuscular administration in cattle. Rev. Sci. Tech..

[23] Chen C.L., Gu X. (1995). Determination of tetracycline residues in bovine milk, serum and urine by capillary electrophoresis. J. AOAC Int..

[24] Craigmill A.L., Miller G.R., Gehring R., Pierce A.N., Riviere J.E. (2004). Meta-analysis of pharmacokinetic data of veterinary drugs using for Food Animal Residue Avoidance Databank: Oxytetracycline and procaine penicillin G. J. Vet. Pharmacol. Ther..

[25] Fritz J.W., Zuo Y. (2007). Simultaneous determination of tetracycline oxytetracycline and 4-epitetracycline in milk by high-performance liquid chromatography. Food Chem..

[26] Khaskheli M., Malik R.S., Arain M.A., Soomro A.H., Arain H.H. (2008). Detection of B-Lactam Antibiotic Residues in Marker Milk. Pak. J. Nutr..

[27] Aalipour F., Mirlohi M., Jalali M. (2013). Prevalence of antibiotic residues in commercial milk and its variation by season and thermal processing methods. Int. J. Environ. Health Eng..

[28] Chowdhury S., Hassan M.M., Alam M., Sattar S., Md Bari S., Saifuddin A.K.M., Hoque M.A. (2015). Antibiotic residues in milk and eggs of commercial and local farms at Chittagong, Bangladesh. Vet. World..

[29] Khosrokhavar R., Hosseini M.J., Amini M., Pirali-Hamedani M., Ghazi-Khansari M., Bakhtiarian A. (2008). Validation of an analytical methodology for determination of oxytetracycline residue in milk by HPLC with UV detection. Toxicol. Mech. Methods.

[30] Boultif L., Zeghilet N., Chebira B., Agabou A., Mekroud A. (2014). Validation of a high performance liquid Chromatography (hplc) method for the determination of oxytetracycline residues in milk. Adv. Anim. Vet. Sci..

[31] Kamel A.M., Fouda H.G., Brown P.R., Munson B. (2002). Mass spectralcharacterization of tetracyclines by electrospray ionization, H/D/exchange, and multiple stage mass spectroametry. J. Am. Soc. Mass Spectrom..

[32] Pijpers A., Schoevers E.J., van Gogh H., van Leengoed L.A., Visser I.J., van Miert A.S., Verheijden J.H. (1990). The pharmacokinetics of oxytetracycline following intravenous administration in healthy and diseased pigs. J. Vet. Pharmacol. Ther..

[33] Hall W.F., Kniffen T.S., Bane D.P., Bevill R.F., Koritz G.D. (1989). Plasma concentrations of oxytetracycline in swine after administration of the drug intramuscularly and orally in feed. J. Am. Vet. Med. Assoc..

[34] Nielsen P., Gyrd-Hansen N. (1996). Bioavailability of oxytetracycline tetracycline and chlortetracycline after oral administration to fed and fasted pigs. J. Vet. Pharmacol. Ther..

[35] Gajda A., Nowacka-Kozak E., Gbylik-Sikorska M., Cybulski P. (2022). UHPLC-MS/MS Analysis of Antibiotics Transfer and Concentrations in Porcine Oral Fluid after Intramuscular Application. Pharmaceuticals.

[36] Gerosa S., Skoet J. (2012). Milk Availability Trends in Production and Demand and Medium-Term Outlook. https://www.fao.org/3/an450e/an450e00.pdf.

[37] (2018). Maximum Residue Limits (MRLs) and Risk Management Recommendations (RMRs) for Residues of Veterinary Drugs in Foods. CX/MRL 2-2018. https://www.fao.org/fao-who-codexalimentarius/sh-proxy/en/?lnk=1&url=https%253A%252F%252Fworkspace.fao.org%252Fsites%252Fcodex%252FStandards%252FCXM%2B2%252FMRL2e.pdf.

[38] Schild S.L., Foldager L., Rangstrup-Christensen L., Pedersen L.J. (2020). Characteristics of Piglets Born by Two Highly Prolific Sow Hybrids. Front. Vet. Sci..

[39] Theil P.K., Nielsen T.T., Kristensen N.B., Labouriau R., Danielsen V., Lauridsen C., Jakobsen K. (2002). Estimation of milk production in lactating sows by determination of deuterated water turnover in three piglets per litter. Acta Agric. Scand. Sect. A-Anim. Sci..

